# Erysipelas and Pyæmia in Relation to Surgical Accidents, Treated by Wine, Ammonia, and Other Stimulants

**Published:** 1855-10

**Authors:** 


*Erysipelas and Pyæmia in Relation to Surgical Accidents, treated by wine, ammonia, and other stimulants.* (Under the care of DR. TODD in King's College Hospital.)

The prophylaxis or due prevention of purulent infection, in surgical cases in hospitals, must ever be a subject of paramount interest to the practising surgeon. Pyæmia holds a very prominent place in the causes of death in those operated on in hospitals. An opinion, however, has gained ground, perhaps too readily, of the total incorrectness of this assertion, and patients have been too often abandoned to their fate under this impression. The rule usually adopted amongst surgeons is to prevent as much as possible the formation of pus, or, where pus is already formed, to assist and encourage its exit by free incisions and pressure over purulent sinuses. We have been very much gratified, however, of late, with the result of some cases allied to pyæmia, under the care of Dr. Todd, in the medical wards of King's College Hospital. Dr. Todd proposes to check pyæmia by diminishing excess of inflammation in wounds, and checking erysipelas, both of which, as leading to the secondary results of inflammation, help to induce pyæmic fever. Pyæmia amongst the wounded soldiers, in summer, in the Crimea, for instance, is not less fatal than scorbutus in the winter, but both are curable, according to Dr. Todd, if only treated by stimulants from the first, such as brandy, beef-tea, ammonia, opium, &c. It would seem, that while surgeons have been in too many cases looking on pyæmia as theoretically quite incurable, it has often cured itself; and while we have been hitherto absorbed in the microscopical question whether pus-globules enter the circulation in this disease as such or are broken up into poisonous matter, the more practical men have recognized phlebitis as at once an almost constant cause of pyæmia, and a not unfrequent mode of *cure* also of the pyæmic complication, set up by Nature itself. The cause of pyæmia, we may say, as when not suspected, the mixture of the elements of pus in phlebitis, if not pus itself, may exist along the veins, even as far back as the auricles of the heart. This phlebitis, if properly treated, leads to a blocking up of the vein by fibrinous adhesion and a stoppage of the purulent absorption, thus becoming a means of cure; but if neglected or encouraged by depressing or antiphlogistic remedies, only leading to the most disastrous results—further formation of pus, with hectic and irritative fever, general debility of the system, unfavorable to the dispersion or resolution of the fever, or poison in the system, and death.

One of Dr. Todd's cases, a type of a class of cases received into this and other hospitals, was the following:—

J. C——, aged about 20, admitted into King's College Hospital with bad erysipelas of the face and head. The case was one of those very severe attacks in which the man might die, and the erysipelas might be said to have attacked the brain. Dr. Todd, however, believes that erysipelas does not "fly to the brain," but that, as in the puerperal peritonitis of women, which he considers as an erysipelatous affection, as in œdema of the glottis, and as in pyæmia, both so fatal, the cause of death is to be found too often in the secondary results of inflammation and fever. Erysipelas has a tendency to attack the areolar cellular tissue of organs, but this, according to Dr. Todd, is altogether obviated by treating such cases from the beginning with beef-tea and ammonia and bark, and not, as formerly, by leeches, bleeding, and mercurial purgatives. Erysipelas may thus be cut short, and get well in seven days, instead of being eighteen or twenty. We have observed in all the hospitals, of late, these views coming more and more into use. Some of the very old practitioners still adhere to water-gruel, purgatives, depletion, and antiphlogistics; while as a more general result the opposite plan leads to a more scientific treatment of cases and to the saving of human life.

Pyæmia is sometimes checked by stopping the inflammation of the veins by the pre-application of caustic, or as more recently advised and practised, by that of the actual cautery itself along the course of the vein, to encourage adhesive inflammation. The experiments of Cruveilhier, in which mercury was injected into the veins, and almost simulated the phenomena of some of the secondary results of inflammation and pyæmia, all further corroborate Dr. Todd's opinions. Lebert, it is true, demonstrates that pus in the blood destroys the globules of the blood itself, diminishing its fibrine and precipitating its fatty matters; but not showing that this may not lead to purulent deposits in the lungs and liver, ending in death. These fatty deposits in the heart and blood-vessels of those dying of pyæmia we have seen in the dead house; but all or many of such secondory results of inflammation attending surgical operations may be obviated, according to Dr. Todd, by joining a medical to a surgical treatment of such cases. The remote causes of pyæmia and erysipelas, according to the most truthful observers, being the crowding together of surgical patients and neglect of their wounds; the closing up too much of wounds and stumps as observed to us not long since by Sir Robert Carswell; next, wounds of veins and ligatures of veins; but above all, certain epidemic or meteorological influences, especially in large cities like Paris or London, which at specific seasons interfere with the healthy formation of blood and pus; while the immediate or efficient pathological cause of pyæmia seems undoubtedly to be pus or its serum absorbed into the current of the circulation. Thus the surgeons of the French school do not hesitate, by amputation of a limb, to cut off the absorbing surface on the access of the first rigors of the pyæmic fever; while Dr. Todd, on the other hand, would obviate it on the principle of "principiis obsta," anticipating the secondary evils of inflammation by cutting the latter short by the treatment now found more effectual than that formerly observed.—*Lancet.*

